# Prevalence of Hepatitis Delta Virus (HDV) Infection in Chronic Hepatitis B Patients with Unusual Clinical Pictures

**DOI:** 10.5812/hepatmon.6731

**Published:** 2013-08-16

**Authors:** Shiva Ghamari, Seyed Moayed Alavian, Mario Rizzetto, Antonella Olivero, Antonina Smedile, Abulfazl Khedive, Seyed Ehsan Alavian, Mohammad Reza Zolfaghari, Seyed Mohammad Jazayeri

**Affiliations:** 1 Hepatitis B Molecular Laboratory, Department of Virology, School of Public Health, Tehran University of Medical Sciences, Tehran, IR Iran; 2 Baqiyatallah University of Medical Sciences, Baqiyatallah Research Centre for Gastroenterology and Liver Disease, Tehran, IR Iran; 3 Middle East Liver Diseases Center, Tehran, IR Iran; 4 Department of Gastroenterology and Hepatology, San Giovanni Battista University Hospital (Molinette), Turin, Italy; 5 Department of Microbiology, Islamic Azad University, Qom, IR Iran

**Keywords:** Hepatitis Delta Virus, Prevalence, Antibodies

## Abstract

**Background:**

Probably 5% of the HBV carriers have HDV super infection. The risk of fulminant hepatitis, cirrhosis and hepatocellular carcinoma is higher in superinfection than the settings when HBV is alone.

**Objectives:**

The aim of this study was to evaluate the prevalence of HDV in Iranian HBV isolates and to compare their clinical and virological pictures as well as their HDV genetic variations with other worldwide isolates.

**Patients and Methods:**

81 carriers with positive results for HBsAg with upper limit ranges of ALT and low or undetectable levels of HBV viral load who did not respond to HBV therapy were selected. After RT amplification of HDV Delta antigen, direct sequencing and phylogenetic study were performed to explore the genotype(s) and nucleotide/amino acid variations.

**Results:**

12 (14.8%) patients had positive results for both HDV RNA and anti-HDV. The mean ALT level was higher in HDV positive patients (75.9 U/ML) than HBV-mono-infected individuals; however, the mean HBV viral load was lower in coinfected patients than HBV-mono-infected patients. Phylogenetically, genotype I was the only detected genotype, and the most closely related isolates were of Turkish, Italian and Mongolian origin. Within the delta Ag, there were 326 nucleotide mutations, of which 111 and 215 were silent and missense, respectively. The total number of amino acid substitution was 148; most were located in known functional/epitopic domains. There was no correlation between the numbers of amino acid mutations, with clinical, virological status of the patients.

**Conclusions:**

HDV should be suspected in HBV carriers with unusual clinical and virological pictures. Relatedness of Iranian HDV isolates to Italian and Turkish sequences proposed a common Caucasian origin for the distribution of HDV genotype I in this ethnic group.

## 1. Background

Up to 5% of the world’s population have been infected with hepatitis B virus (HBV), of whom probably 5% of the HBV carriers have hepatitis D virus (HDV) superinfection. It is estimated that 18 million people are infected with HDV world wild. Hepatitis D virus (HDV) is a defective single-stranded RNA virus which requires the HBsAg of HBV to establish infection in humans. The antigenomic strand of HDV encodes the only protein, hepatitis delta antigen (HDAg), in two molecular-weight forms. The large form carries an extra 19-amino-acid (aa) extension at the C terminus which plays a key role in the packaging of HDV and suppresses viral replication in a trans-dominant-negative manner, while the small HDAg plays an essential role in transactivating the replication of the HDV RNA (1). The disease spectrum of HDV infection varies greatly from fulminant hepatitis, rapidly progressive disease, to a subclinical course. Persistent replication of HDV associated with continuous hepatic inflammation and elevated alanine aminotransferase (ALT) levels is a characteristic of chronic active hepatitis D (2).

There are two modes of the HDV infection: coinfection, results from acute infection with both hepatitis B virus (HBV) and HDV, whereas superinfection results from HDV infection of patients with underlying chronic hepatitis B infection. Super infection with HDV increases higher risk of chronic HBV infection leads to progressive disease and cirrhosis in approximately 80% of cases than coinfection with HBV and HDV (3).

Based on phylogenic analysis, HDV isolates collected worldwide have been classified into 8 groups, HDV-1 (former genotype I), HDV-2 (former genotype IIa), HDV-3 (former genotype III), HDV-4 (former genotype IIb), and HDV-5 to HDV-8 (4). These HDV types show different geographic distribution and are associated with different disease patterns. Genotype 1 has been distributed worldwide, whereas other HDV genotypes circulate unevenly. The prevalence of HDV in Iranian hepatitis B infected individuals reported to be between 2.5% and 5.8% (5-7), and the major genotype was genotype I (8, 9).

## 2. Objectives

The aim of this study was to evaluate the prevalence of HDV in Iranian HBV isolates and to compare their clinical and virological pictures as well as their HDV genetic variations with other worldwide isolates.

## 3. Patients and Methods

### 3.1. Patients Design

81 patients with HBV infection, who were admitted to the Tehran Hepatitis Center (THC), Iran, for more than two years and who agreed to enter the investigation included in the study. Patients were chosen by the following criteria: positive results for HBs Ag, HBeAg negative chronic hepatitis or cirrhosis confirmed by histological and clinical evaluation, zero to low levels of HBV DNA, raised levels (5- 50 fold) of aspartate amino transferase (AST) and alanine amino transferase (ALT), no evidence of HIV or HCV coinfection, and no evidence of alcohol intake or exposure to hepatotoxic drugs. All patients had received IFN for two years. Informed consents were taken from patients.

### 3.2. RNA Extraction

Total RNA was extracted using acid guanidine phenol-chloroform method. In brief, 50µl of serum patient was mixed with lyses buffer (Qiagen), Proteinase K (Qiagen) RNA Carrier (Invitrogen) was incubated overnight. In aqueous phase, RNA was extracted by chloroform, and then the RNA was precipitated with isopropanol and washed twice with ethanol, dissolved in 200 µl of water, and stored at -80˚C until used.

### 3.3. Synthesis of cDNA for HDV RNA RT-PCR

9 µl of RT mixture, dNTP (10 mmol) (Roche), DTT (Roche, Germany), RT Enzyme (Invitrogen, Germany) and RNase (Promega, Germany) were transferred to 1.5 µl extracted RNA. Transcription was set up on ice and was performed as following thermal cycling: 25 ˚c for 5 minutes, 50 ˚c for 35 minutes, and 95 ˚c for 2 minutes. Subsequently, 10.5 µl of final cDNA solution was obtained from this reaction.

### 3.4. PCR Analysis of Hepatitis D Virus

Qualitative detection of HDV-RNA in serum was performed by single nested PCR amplification of a highly conserved region of HDV genome as described previously ( 10 ). For standard PCR, the region 1 (nucleotides 886 to 1288) corresponded to the C-terminal portion of the hepatitis delta Ag (HD Ag) coding region and included the RNA editing site and the poly adenylation signal (Smedile, et al. unpublished). The primers were designed according to an Italian HDV-infected genotype I (Table 1). Both single and nested PCR were performed by hot star Taq (Qiagen, Germany). The conditions of single PCR using primers EAB235 and EAB 237 set were: denaturation at 94˚C for 30 seconds, annealing at 64˚C for 1 min, and extension at 72˚C for 1 min for 40 cycles. The firs denaturation was performed at 94˚C for 2 min, and the last extension was performed at 72˚C for 10 min. 

**Table 1.** Oligonucleotide Primers used for RT-PCR and Sequencing

	Sequence
**First Round PCR**	
PrimerEAB 235 HDV (858)	5' GCC CAG GTC GGA CCG CGA GGA GGT 3’
primerEAB 237 HDV (1312)	5' ACA AGG AGA GGC AGG ATC ACC GAC 3’
**Second Round PCR**	
primer EAB 236 HDV (883)	5' GAA GGA AGG CCC TCG AGA ACA AGA 3’
primer EAB 238 HDV (1288)	5' GAG ATG CCA TGC CGA CCC GAA GAG 3’

### 3.5. Quantitative Determination of HDV-RNA and HBV-DNA

Serum HBV-DNA levels were determined by a commercial quantitative PCR assay (Amplicor HBV monitor test, Roche Diagnostics, GmbH Mannheim, Germany) with a sensitivity threshold of 2500 copies/ml (78UI/ml). HBV genotypes were determined based on reverse hybridization line probe assay.

### 3.6. Biochemistry and Serologic Tests

ALT, AST, alpha-fetoprotein, direct and total bilirubin, albumin, and prothrombin time were assessed by standard methods. Serologic markers of HBV, HAV and HCV were tested by commercial enzyme immunoassays (Abbot Laboratories, North Chicago, IL; Sorin Biomedica, Saluggia, Italy). Competitive enzyme immunoassay (ELISA) kit (DIA.PRO, Milano, Italy) was used for the detection of antibodies to hepatitis delta.

### 3.7. Sequencing

Amplified PCR products were purified by EXOSAP (USB Corporation, Cleveland, Ohio, USA) and obtained samples were directly sequenced by an automated sequencer (BMR-GENOMICS, Padova, Italy), using internal primers EAB 236 and EAB 238.

### 3.8. Mutational and Phylogenetic Analysis

Sequencing chromatograms were analyzed by Chromas (Chromas, version 2.3, Technelysium Pty Ltd, Tewantin, Australia) and Bioedit (5.0.9-tom Hall, Department of Microbiology, North Carolina state university) software. The alignment of HDAg amino acid sequences were performed by Clustal W (version 1.8-ftp.ebi.ac.uk). Final edited sequences were subjected for phylogenic analysis by applying Mega4 (version 4.1).

### 3.9. Indirect Immune Fluorescent Assay

Indirect immunofluorescent assay was used on slides of mouse tissue (kidney, liver, stomach) to evaluate the main antibodies: ANA (anti-nuclear antibodies), ASMA (anti-smooth muscle antibodies), LKM (liver-kidney microsomes antibodies), ARA (anti-reticulin antibodies), AMA (anti-mitochondrial antibodies), anti-ribosomal antibodies, APCA (anti-parietal cell antibodies), and ABBA (anti-brush border antibodies). Serum samples were diluted 1:40 in PBS. A polyclonal rabbit anti-human IgA, IgG, IgM, Kappa, Lambda/FITC was used as fluorescent conjugate. On each slide tested, a negative control (IFA System Negative Control, NOVA Lite ANA KSL, INOVA Diagnostics), and a positive internal control (ANA homogenous pattern) were also tested in each essay.

## 4. Results

Of 81 individuals studied, 21 patients were female (25.9%) and 60 patients were male (74.1%), the mean age was 42. All of patients were of Iranian origin with different ethnic groups. Of the 81 serum samples tested with positive results for HBs-Ag, 12 (14.8%) patients had positive findings for both anti-HDV and HDV RNA (Table 2), of whom 5 and 7 were male and female, respectively. All had positive results for anti-HDV IgG as evidence of past infection, whereas, 8 had positive findings for anti-HDV IgM, suggestive of recent infection. The results revealed that all 12 isolates contained HBV genotype D and subgenotype D1. The details of patients with positive results for HDV-RNA are summarized in Table 2. All patients were HBs-Ag positive HBeAg negative with mean ALT and AST levels of 38.44 (IU/L) and 30.79 (IU/L), respectively. However, in patients who had positive results for HDV, the mean values for ALT level were 88.30 (IU/L) (kurtosis = 1.33) and 74.8 for AST (IU/L) (kurtosis = 1.33). All patients had a very low to moderate HBV DNA levels (results not shown); however, all HDV-positive samples showed very low levels (negative to 15 copy/ml) of HBV DNA (Table 2). 

**Table 2.** Summary of Demographic, Clinical and Virological Characteristics of Patients With Positive Results for HBV and HDV Viral Load Levels are Indicated as copy/mL

Number	Sex	Age	ALT	AST	HBV Viral Load	HDV viral load, copy/ml	Anti-HDV IGM	Anti-HDV IGG	HDV- PCR	LIVER Biopsy	Number of Amino Acid Mutations	IF assay
**2**	f	45	103	65	< 15	1.45 E+08 145000000	positive	positive	positive	cirrhosis	12	ANA+ 1:40 homogenous
**4**	f	56	45	42	NEGATIVE	2.05 E+06 2050000	negative	positive	positive	HCC	11	ANA+ 1:40 speckled
**5**	m	50	87	83	NEGATIVE	1.47 E+06 1470000	positive	positive	positive	chronic Hepatitis B	15	ABBA+
**6**	f	48	37	32	2673	3.62 E+07 36200000	positive	positive	positive	cirrhosis	8	ANA+ > 1:40 speckled
**7**	f	40	268	238	< 15	3.84 E+07 38400000	positive	positive	positive	Chronic Hepatitis B	12	ANA+ 1:80 homogenous
**8**	f	58	90	73	NEGATIVE	2.17 E+08 217000000	positive	positive	positive	13 cirrhosis	13	ASMA+ > 1:80, ABBA+
**9**	f	58	38	30	240	5.28 E+05 528000	negative	positive	positive	16 cirrhosis	15	Negative
**10**	m	62	90	113	< 15	1.38 E+05 138000	negative	positive	positive	cirrhosis	11	ANA + 1:80 homogenous
**12**	m	53	24	47	NEGATIVE	1.29 E+06 1290000	positive	positive	positive	chronic Hepatitis B	22	ANA + 1:40 fine speckled
**13**	m	57	101	25	NEGATIVE	2.66 E+06 2660000	positive	positive	positive	cirrhosis	9	ANA+ > 1:80 fine speckled, ASMA + > 1:80
**16**	f	53	43	54	344	1.44 E+05 144000	negative	positive	positive	chronic Hepatitis B	11	ABBA+
**84**	f	49	35	42	<15	8.69 E+08 869000000	positive	positive	positive	Chronic Hepatitis B	9	ANA+ 1:80 homogenous

### 4.1. Indirect Immunoflurescent

**Figure 1.**
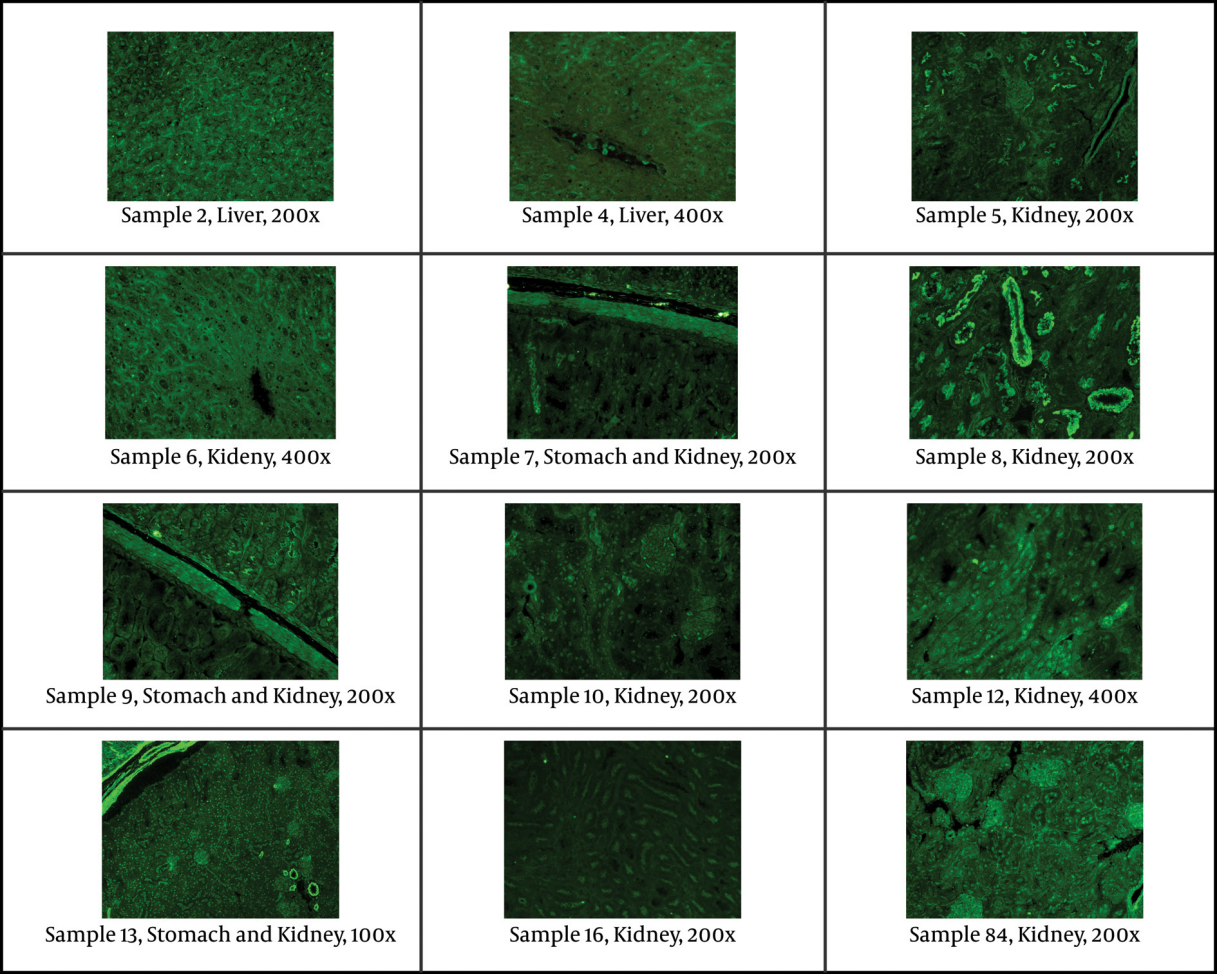
Staining of Antinuclear Antibody (ANA), ABBA (anti-brush border antibody) and Smooth Muscle Antibody (ASMA) in Different Tissues of Patients With Positive Results for HDV Visualized by Indirect Immunofluorescence. The Concentration Cut off for Positivity of Antibodies as Well as the Magnification of Individual Samples Is Included.

12 HDV-positive samples were studied by indirect immunoflurescent on mouse tissue for different main autoantibodies. All samples but one (sample No 9) had positive results for at least one autoantibody with different concentration and distribution patterns (Figure 1 and Table 2). Seven samples had positive results for ANA, and samples 8 and 13 had positive results for two autoantibodies for each (Figure 1 and Table 2). 

### 4.2. Phylogenetic Analysis

**Figure 2.**
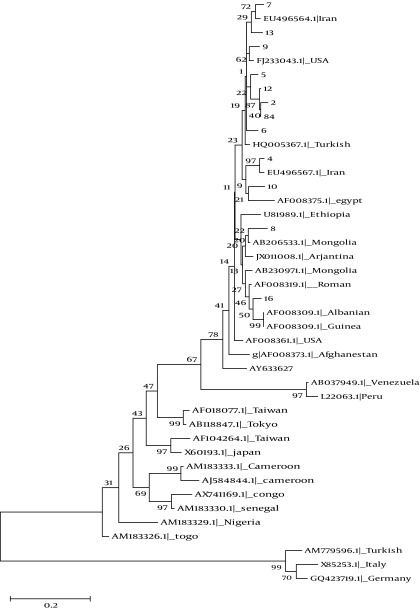
Neighbor Joining Phylogenetic Trees of 12 Samples Along With Reference Sequences of HDV Belonging to Different Clades (1 to 8) Derived From GenBank, Indicated by Their Accession Numbers and Country of Origin. The Sequences Determined in the Study Are Given by the Isolate Code. Note: The bootstrap consensus tree inferred from 1000 replicates is taken to represent the evolutionary history of the taxa analyzed.

A 405- bp fragment of delta antigen taken from each of 12 patients with positive results for HDV-RNA was used for molecular study. The fragments were compared to the 29 reference gene sequences of HDV genotype 1 to 8 available in GenBank database (Figure 2). Genotype Ι was the only detected genotype. 10 of 12 isolates were branched into two separate clusters, and only two isolates (8 and 16) were grouped together with other genotype I sequences (Figure 2).

### 4.3. Mutation Analysis

**Figure 3.**
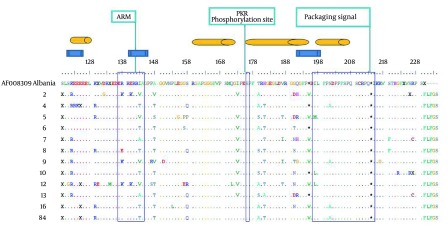
Alignment of Amino Acid Sequences of Partial Delta Ags Encompassing the Specific Functional Domains and Immune Epitopes. Mutations Identified in 12 Sera Using Bio Edit Software Are Illustrated Note: The genotypic, functional, immune epitope domains are depicted in blue rectangle, open bars and orange cylinders, respectively. Variations along the partial small/large delta Ags were shown by letters in comparison to the reference sequence from a genotype I obtained from GenBank (accession number AF008309) from Albania. Dot indicates identity to the reference sequence.

A genotype I isolate from Albania (Accession number AF008309) was chosen for sequencing alignment, due to the less average variations compared to other genotype I in the database sequences. There were 326 nucleotide mutations, of which 111 and 215 were silent and missense, respectively (results not shown). The ratio between silent and missense nucleotide mutations was 0.52. The total number of amino acid substitution was 148 (Figure 3). Within the region known for RNA binding domain (97-146), 18 amino acid mutations were occurred (Figure 3). Within virus assembly domain (195-204), four sequences contained stop codon mutations in position 195, on the other hand eight contained tryptophan (W), indicating expression of large delta antigens in the latter. Within the 19 C-terminus region of Delta Ag, seven point mutations were found (Figure 3). No mutation was found in PKR (Protein Kinase R) phosphorylation site (Figure 3). 

72 mutations were distributed within four known different immune epitopes, of which 52 (72.2%) were occurred as a hotspot domain between amino acid residues 174 and 195 (Figure 3).

### 4.4. Correlation Between Clinical and Virological Data

Overall, the mean ALT level was higher in patients with positive results for HDV (75.9 U/ML) than HBV-mono-infected individuals (results not shown). On the other hand, all HDV-positive cases but one showed a gradient decrease in the median HBV DNA load (from zero to 15 copy ml) compared to HBV mono-infected patients. However, there was no association between the grade of hepatic injury and HDV viral load. In mutational analysis, there were no significant correlations between the number and distribution of mutations and clinical, serological and virological details of patients (Table 2). 

## 5. Discussion

In the present study, we evaluated the clinical and virological courses of HDV infection in a group of chronic HBV patients who showed a mild to moderate grades of hepatic injury, low to undetectable levels of HBV DNA, and a mild increase in the levels of liver enzymes. Biopsy-proven cases of cirrhosis and HCC also included in the patients-studied. While all the studied patients contained different low levels of HBV DNA, all HDV-positive cases but one showed a gradient decrease in the median HBV DNA load (from zero to 15 copy ml). This finding confirms the previous results (11) highlighting the suppression effect of HDV on HBV replication in these patients (12, 13). On the other hand, we could not find any association between HDV RNA load and the severity of liver disease including cirrhosis and HCC, the finding that was reported previously from Pakistan with the same distribution of HBV genotype D like Iran (14).

All samples but one (sample No 9) had positive results for at least one autoantibody with different concentration and distribution patterns. There are some reports in the literatures that HDV infection has been associated with autoimmune phenomenon (15, 16). However, the data that support this hypothesis is based on similar findings. The mechanisms and the significance of such autoantibody production have not been completely understood. Our result together with other results from Iranian studies showed that all HDV-positive cases had genotype I (9). Unpublished data from our lab indicated that all available Iranian HDV isolates have been closely related to European sequences. All these ethnic groups contained HBV genotype D dominantly, and all are of Caucasian origin. The distribution of HDV genotype I in Caucasian suggested that this ethnic group might have infected by the same HBV genotype (type D), then acquired HDV infection with the same genotype (type I) during colonization. Subsequently, due to intermixing between infected people and due to the random genetic drift, each virus undergone multiple variations. Previous finding might confirm this issue as data obtained from Turkey (17, 18), Lebanon (19), Russia (20), Mediterranean Basin and Europe (10, 21, 22), Mongolia (23, 24) and Tajikistan (25) which have shown the predominance of genotype I in genotype D infected HBV patients. The latter hypothesis is based on the finding of amino acid substitutions in residues involved in either immune epitopes and/or functional domains which are variants and not mutations. These positions also contained signature sequences regions specific for genotyping assignment (21, 26).

Patients with HBV/ HDV coinfection may have a more severe acute disease and in approximately 80% of these individuals the chronic HDV infection progresses to cirrhosis within 5-10 years (27). This observation is also noted in our HBV/HDV coinfection patients, as cases of HCC and cirrhosis were seen dominantly in HBV/HDV confection individuals as compared to HBV mono-infection cases. Worldwide, HDV genotype I has been associated with a highly pathogenic potential (28). This issue as well as the impact of HDV on the progression of liver disease in chronic HBV patients underscores the significance of diagnosis and genotyping of HDV in these patients to avoid such complications.

The ratio between silent and missense nucleotide mutations was 0.52, indicating a positive selection pressure exerted on these antigens. Among the four known B cell epitope residues (29) (Figure 3), 174-195 contained a hot spot motif for variation, as our results showed that it was covariated in all patients. Other epitopes showed only a few mutations in isolates. Due to the divergent structure of C-terminus domain of delta Ag (30), these variants seem to be the consequence of selection pressure imposed by host HLA during a long term process, which is different between versatile ethnic groups (31).

In the present study, the number of mutations in C-terminus part of Large-DAg (amino acid 195-214) was low (7 out of 148, 0.04%). Not surprising, as there are three conserved functional domains including nuclear exporting signal, clathrin heavy chain interacting domain and the isoprenylation signal in this short peptide region (32). Furthermore, several regions of RNA-binding sites and sequences in the glycine-prolyne rich segment (160-169, 175-179 and 183-187) are highly conserved among the HDV genotypes (17, 33). We found only two residues (179 and 187) in these domains which contained several mutations. Overall, of 10 residues which were contained mutations, all were contained in regions specific for genotypic classification and immune epitope function and replication (4, 34).

In conclusion, despite a high prevalence of 14.8% of HDV positive cases among chronic HBV carriers in our study, the limited number of samples evaluated and the cross-sectional study design prevented us to draw definitive conclusions. However, the initial suspension based on a florid HBsAg positivity and lack of HBV replicative markers underscore the importance of HDV diagnosis as an underlying disease which could accelerate progression of liver injury in these patients.

## References

[1] Taylor JM (2006). Hepatitis delta virus.. Virology..

[2] Smedile A, Rizzetto M, Gerin JL (1994). Advances in hepatitis D virus biology and disease.. Prog liver dis..

[3] Smedile A, Verme G, Cargnel A, Dentico P, Opolon P, Vergani D (1982). Influence of delta infection on severity of hepatitis B.. The Lancet..

[4] Radjef N, Gordien E, Ivaniushina V, Gault E, Anaïs P, Drugan T (2004). Molecular phylogenetic analyses indicate a wide and ancient radiation of African hepatitis delta virus, suggesting a deltavirus genus of at least seven major clades.. J Virol..

[5] Ghadir M-R, Belbasi M, Heidari A, Sarkeshikian SS, Kabiri A, Ghanooni AH (2012). Prevalence of hepatitis d virus infection among hepatitis B virus infected patients in qom province, center of iran.. Hepatitis monthly..

[6] Rezvan H, Taroyan S, Forouzandeh B, Fadaiee S, Azordegan F (1990). A study on delta virus infection and its clinical impact in Iran.. Infection..

[7] Roshandel G, Semnani S, Abdolahi N, Besharat S, Keshtkar A-A, Joshaqani H (2008). Prevalence of hepatitis D virus infection in hepatitis B surface antigen-positive subjects in Golestan province, northeast Iran.. J Microbiol Immunol Infect..

[8] Behzadian F, Sabahi F, Karimi M, Sadeghizadeh M, Maghsoudi N, Forooshani RS (2005). Molecular phylogenetic analysis of Iranian HDV complete genome.. Virus Genes..

[9] Mohebbi SR, Zali N, Derakhshan F, Tahami A, Mashayekhi R, Amini-Bavil-Olyaee S (2008). Molecular epidemiology of hepatitis delta virus (HDV) in Iran: a preliminary report.. J Med Virol..

[10] Niro GA, Smedile A, Ippolito AM, Ciancio A, Fontana R, Olivero A (2010). Outcome of chronic delta hepatitis in Italy: a long-term cohort study.. J Hepatol..

[11] Kiesslich D, Crispim MA, Santos C, Ferreira FdL, Fraiji NA, Komninakis SV (2009). Influence of hepatitis B virus (HBV) genotype on the clinical course of disease in patients coinfected with HBV and hepatitis delta virus.. J Infect Dis.

[12] Jardi R, Rodriguez F, Buti M, Costa X, Cotrina M, Galimany R (2001). Role of hepatitis B, C, and D viruses in dual and triple infection: Influence of viral genotypes and hepatitis B precore and basal core promoter mutations on viral replicative interference.. Hepatology..

[13] Sagnelli E, Coppola N, Scolastico C, Filippini P, Santantonio T, Stroffolini T (2000). Virologic and clinical expressions of reciprocal inhibitory effect of hepatitis B, C, and delta viruses in patients with chronic hepatitis.. Hepatology..

[14] Mumtaz K, Ahmed US, Memon S, Khawaja A, Usmani MT, Moatter T (2011). Virological and clinical characteristics of hepatitis delta virus in South Asia.. Virol J..

[15] Meşe S, Ozekinci T, Atmaca S, Arikan E, Akin D (2008). Investigation of anticardiolipin antibodies in chronic hepatitis B infection together with total anti-delta positivity].. Mikrobiyoloji bülteni..

[16] Strassburg CP, Obermayer-Straub P, Alex B, Durazzo M, Rizzetto M, Tukey RH (1996). Autoantibodies against glucuronosyltransferases differ between viral hepatitis and autoimmune hepatitis.. Gastroenterology..

[17] Shakil AO, Hadziyannis S, Hoofnagle JH, Di Bisceglie AM, Gerin JL, Casey JL (1997). Geographic distribution and genetic variability of hepatitis delta virus genotype I.. Virology..

[18] Yurdaydin C, Bozkaya H, Gürel S, Tillmann HL, Aslan N, Okcu-Heper A (2002). Famciclovir treatment of chronic delta hepatitis.. J Hepatol..

[19] Ramia S, El-Zaatari M, Sharara AI, Ramlawi F, Farhat B (2007). Current prevalence of hepatitis delta virus (HDV) infection and the range of HDV genotypes in Lebanon.. Epidemiol Infect.

[20] Flodgren E, Bengtsson S, Knutsson M, Strebkova EA, Kidd AH, Alexeyev OA (2000). Recent high incidence of fulminant hepatitis in Samara, Russia: molecular analysis of prevailing hepatitis B and D virus strains.. J Clin Microbiol.

[21] Zachou K, Yurdaydin C, Drebber U, Dalekos GN, Erhardt A, Cakaloglu Y (2010). Quantitative HBsAg and HDV‐RNA levels in chronic delta hepatitis.. Liver Int..

[22] Bielawski KP, Zietkowski D, Charmuszko U, Sikorska K, Stalke P (2006). Hepatitis delta virus infection in chronically HBV-infected patients from northern Poland.. Arch virol.

[23] Tsatsralt‐Od B, Takahashi M, Endo K, Buyankhuu O, Baatarkhuu O, Nishizawa T (2006). Infection with hepatitis A, B, C, and delta viruses among patients with acute hepatitis in Mongolia.. J Med Virol..

[24] Tsatsralt-Od B, Takahashi M, Nishizawa T, Endo K, Inoue J, Okamoto H (2005). High prevalence of dual or triple infection of hepatitis B, C, and delta viruses among patients with chronic liver disease in Mongolia.. J Med Virol..

[25] Khan A, Kurbanov F, Tanaka Y, Elkady A, Sugiyama M, Dustov A (2008). Epidemiological and clinical evaluation of hepatitis B, hepatitis C, and delta hepatitis viruses in Tajikistan.. J Med Virol..

[26] Casey JL, Gerin JL (1998). Genotype-specific complementation of hepatitis delta virus RNA replication by hepatitis delta antigen.. J Virol.

[27] Fattovich G, Boscaro S, Noventa F, Pornaro E, Stenico D, Alberti A (1987). Influence of hepatitis delta virus infection on progression to cirrhosis in chronic hepatitis type B.. J Infect Dis.

[28] Su CW, Huang YH, Huo TI, Shih HH, Sheen I, Chen SW (2006). Genotypes and viremia of hepatitis B and D viruses are associated with outcomes of chronic hepatitis D patients.. Gastroenterology..

[29] Wang JG, Jansen RW, Brown EA, Lemon SM (1990). Immunogenic domains of hepatitis delta virus antigen: peptide mapping of epitopes recognized by human and woodchuck antibodies.. J virol..

[30] Hughes SA, Wedemeyer H, Harrison PM (2011). Hepatitis delta virus.. The Lancet..

[31] Jazayeri M, Basuni AA, Sran N, Gish R, Cooksley G, Locarnini S (2004). HBV core sequence: definition of genotype‐specific variability and correlation with geographical origin.. J Viral Hepatitis.

[32] Huang C-R, Lo SJ (2010). Evolution and diversity of the human hepatitis D virus genome.. Adv bioinformat.

[33] Langon T, Fillon S, Pichoud C, Hantz O, Trepo C, Kay A (1998). Analysis of a hepatitis delta virus isolate from the Central African Republic.. Res virology.

[34] Anisimova M, Yang Z (2004). Molecular evolution of the hepatitis delta virus antigen gene: recombination or positive selection?. J Mol Evol.

